# Evidence of the Extrahepatic Replication of Hepatitis E Virus in Human Endometrial Stromal Cells

**DOI:** 10.3390/pathogens9040295

**Published:** 2020-04-17

**Authors:** Mohamed A. El-Mokhtar, Essam R. Othman, Maha Y. Khashbah, Ali Ismael, Mohamed AA Ghaliony, Mohamed Ismail Seddik, Ibrahim M. Sayed

**Affiliations:** 1 Department of Medical Microbiology and Immunology, Faculty of Medicine, Assiut University, 71515 Assiut, Egypt; elmokhtarma@aun.edu.eg; 2 Reproductive Science Research Center, Assiut University, 71515 Assiut, Egypt; essamrash@yahoo.com (E.R.O.); mahakhashbah@pg.cu.edu.eg (M.Y.K.); 3 Department of Obstetrics and Gynecology, Assiut University, 71515 Assiut, Egypt; 4 Department of Reproductive Medicine, Academic Endometriosis Center, Amsterdam University Medical Center, Postbus 22660, 1100 DD Amsterdam, The Netherlands; 5 Department of Internal Medicine, Faculty of Medicine, Zagazig University, 44519 Zagazig, Egypt; Menna_rana@yahoo.com; 6 Department of Tropical Medicine and Gastroenterology Department, Assiut University, 71515 Assiut, Egypt; ghal_m@yahoo.com; 7 Department of Clinical Pathology, Faculty of Medicine, Assiut University, 71515 Assiut, Egypt; moh.ismail310@aun.edu.eg; 8 Department of Pathology, School of Medicine, University of California, San Diego, CA 92093, USA

**Keywords:** HEV, endometrial stromal cells, pathogenesis, extrahepatic replication, female genital system, pregnancy, ribavirin

## Abstract

Hepatitis E virus (HEV) is the most common cause of acute viral hepatitis worldwide. The tropism of HEV is not restricted to the liver, and the virus replicates in other organs. Not all the extrahepatic targets for HEV are identified. Herein, we found that non-decidualized primary human endometrial stromal cells (PHESCs), which are precursors for the decidua and placenta, are susceptible to HEV infection. PHESCs, isolated from healthy non-pregnant women (n = 5), were challenged with stool-derived HEV-1 and HEV-3. HEV RNA was measured by qPCR, and HEV capsid protein was assessed by flow cytometry, immunofluorescence (IF), and ELISA. HEV infection was successfully established in PHESCs. Intracellular and extracellular HEV RNA loads were increased over time, indicating efficient replication in vitro. In addition, HEV capsid protein was detected intracellularly in the HEV-infected PHESCs and accumulated extracellularly over time, confirming the viral assembly and release from the infected cells. HEV-1 replicated more efficiently in PHESCs than HEV-3 and induced more inflammatory responses. Ribavirin (RBV) treatment abolished the replication of HEV in PHESCs. In conclusion, PHESCs are permissive to HEV infection and these cells could be an endogenous source of HEV infection during pregnancy and mediate HEV vertical transmission.

## 1. Introduction

Hepatitis E virus (HEV) is an emerging pathogen in developing and developed countries [1,2]. HEV is the most common cause of acute viral hepatitis worldwide that causes about 70,000 deaths and 3000 stillbirths annually [3]. HEV isolates that infect humans belong to the Orthohepevirus genus of the Hepeviridae family [4,5]. The Orthohepevirus A includes eight genotypes (gts) (HEV-1-HEV-8), of which at least five gts are infectious to humans [2,4,5]. HEV-1 and HEV-2 only infect humans and they are widely distributed in developing countries, where the infection is mainly transmitted by the fecal-oral route [1,2]. While HEV-3, HEV-4, and HEV-7 are zoonotic, and pigs, wild boars, deer, and camels are the main source of infection [6,7,8,9,10]. The infection by these isolates is transmitted by the ingestion of contaminated animal products and by blood transfusion [7,11,12]. Other HEV isolates, such as HEV-5, HEV-6, and HEV-8, are not confirmed as human pathogens [5,13]. The vertical transmission of HEV-1, but not HEV-3, from mother to fetus is documented [14,15,16]. HEV, especially HEV-1 virus, causes serious outcomes and mortality during pregnancy [17,18]. Complications of HEV during pregnancy depend on several factors, such as virus genotypes, viral load, immune status, and hormonal factors [19].

HEV is a positive-sense single-stranded RNA virus, and its genome is about 7.2 kb and includes three overlapping open reading frames (ORF1, ORF2, and ORF3) with a 5′capping and a 3′-poly A-tail. ORF1 encodes nonstructural proteins that are the enzymes required for HEV replication, such as RNA-dependent RNA polymerase, helicase, cysteine protease, and methyltransferase. ORF2 encodes the structural capsid protein (ORF2), and ORF3 encodes a small phosphoprotein that plays a role in virion morphogenesis and egress from the infected cells [1,2,20].

HEV causes acute infection. In addition, chronic infection and extrahepatic manifestations, especially in the kidney, the nervous system, and placenta, were documented [17,21]. The genital system is another target for HEV replication. The HEV-4 virus could cross the blood–testis barrier, causing severe testicular damage and infertility [22,23]. Similarly, HEV replicates efficiently in the ovary of the rabbits, inducing structural and molecular changes in the ovarian tissues and promoting oocyte apoptosis [24]. In addition, HEV replicates in the uterus and placenta of pregnant BALB/c mice before vertical transmission to the fetus [25]. Little is known about the replication of HEV in the endometrium. The endometrium is a highly dynamic tissue, and endometrial stromal cells are a key component of the endometrium. Primary human endometrial stromal cells (PHESCs), which are fibroblast-like cells, undergo several morphological and functional changes under the effect of sex hormones. In the case of pregnancy, these cells are developed to form the decidua, which mediates the placenta formation, while the decidua endometrium lining is shed off in absence of embryo implantation [26].

Previous studies had shown that viruses can infect endometrial stromal cells during their pathogenesis, such as Zika virus, HIV, and human herpesvirus, and these cells could act as vectors and facilitate viral transmission from mother to fetus or during sexual intercourse [27,28]. A recent study showed that HEV could replicate in organ cultures of the maternal–fetus interface, including the decidua basalis and fetal placenta. Besides, HEV replicated efficiently in stroma cells derived from the decidual and placental tissues, causing alterations in the tissue secretome and severe tissue injury [29]. 

To our knowledge, there is no report on the replication of HEV in PHECSs in the absence of pregnancy. Herein, we show that PHECSs derived from the endometrium of non-pregnant women are permissive to HEV infection.

## 2. Results

### 2.1. Isolation and Characterization of PHESCs

**Figure 1:**
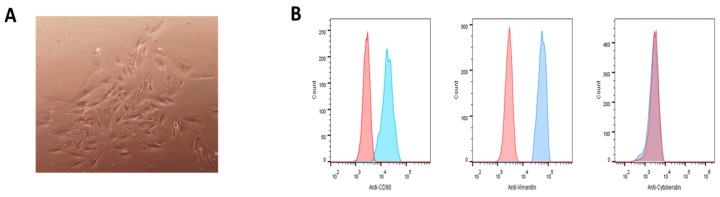
Primary human endometrial stromal cells (PHESCs) were isolated from the endometrium of non-pregnant women. (**A**) The morphological characterization of the isolated PHESCs showing fibroblast like cells. (**B**) The characterization of the isolated PHESCs by staining with FITC-conjugated human anti-CD90 (left), A488-conjugated anti-vimentin (middle), or A488-conjugated anti-cytokeratin (right). The blue curves represent PHESCs stained with these antibodies, respectively, and the red histograms represent the staining of these cells with isotype-matched control antibodies.

PHESCs were isolated from endometrial tissue biopsies collected from healthy non-pregnant women (n = 5), aged 27–35 years, admitted to Assiut University Hospitals, as described in our previous studies [30]. The isolated cells have a characteristic microscopic morphology that confirms that they were fibroblast-like cells (Figure 1A). Analysis of the isolated cells by flow cytometry showed that over 95% of the cells expressed the stromal cell markers (CD90 and vimentin), but they did not express cytokeratin, which is a marker for epithelial cells, confirming that the isolated cells were endometrial stromal fibroblast-like cells (Figure 1B). These results were consistent in all PHESCs isolated from all donors enrolled in this study.

### 2.2. HEV RNA Kinetic in PHESCs Challenged with HEV Preparations

**Figure 2:**
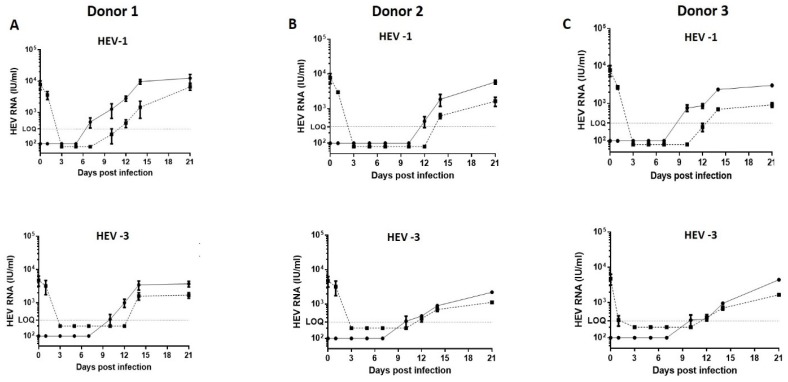
Hepatitis E virus (HEV) RNA kinetic in PHESCs challenged with HEV preparations. PHESCs, isolated from different donors, were challenged with stool-derived HEV-1 (upper panels) or stool-derived HEV-3 (lower panels), then the lysis of cells and/or collection of supernatants were done at 0, 1, 3, 5, 7, 10, 12, 14 and 21 days post-infection. (**A**) represents PHESCs isolated from donor #1, (**B**) represents PHESCs isolated from donor #2, and (**C**) represents PHESCs isolated from donor #3. The HEV RNA load was quantified in these samples by qPCR. The solid line represents intracellular HEV RNA load, the dotted line represents extracellular RNA load, and LOQ is the limit of quantification. Depicted are the mean values of three independent experiments ± SEM.

PHESCs were challenged with the stool-derived HEV-1 and HEV-3 at equal dose (10^6^ IU/ well). In donor # 1, intracellular HEV RNA was started to be detected in PHESCs by day 7 and day 10 post-infection (p.i.) in HEV-1- and HEV-3-infected cells, respectively. Moreover, HEV RNA was detectable in the supernatant of the infected cells at day 12 and day 14 post-infection, respectively. The intracellular viral load increased over time, reaching up to 1.6 × 10^4^ IU/mL and 4.5 × 10^3^ IU at day 21 p.i. in the HEV-1- and HEV-3-infected cells, respectively (Figure 2A). Similarly, the extracellular viral load increased over time, reaching to 7.8 × 10^3^ and 2 × 10^3^ IU/mL at day 21 p.i. in the HEV-1- and HEV-3-infected cells, respectively (Figure 2A). Likewise, the intracellular and extracellular HEV titers were increased in PHESCs isolated from donor #2 and donor #3 and challenged with the same viruses (Figure 2B,C). HEV replicated also in PHESCs isolated from donor #4 and donor #5, but the kinetic of the viral replication was slightly slower in PHESCs derived from those two donors (#4 and #5) compared to the other donors (#1-#3) since the virus started to be detected intracellularly at day 14 p.i. and extracellularly at day 17 p.i. (data not shown). Comparing the propagation of HEV-1 versus HEV-3 in PHESCs revealed that the HEV-1 replicated more efficiently than HEV-3. The mean HEV load in the supernatant of HEV-1-infected PHESCs was higher than the mean viral load in the supernatant of HEV-3-infected cells despite the fact that the inoculums were of equal doses. We used hepatocyte-derived cell line (Huh7.5) cells as a positive control for HEV replication and to compare the replication of HEV in Huh 7.5 cells versus PHESCs. Huh 7.5 cells were challenged with the same inoculums, and, at the same dose, and HEV RNA was monitored. HEV-3, but not HEV-1, replicated efficiently in Huh 7.5 cells and the viral load increased over time (Figure S1). HEV-3 replication was significantly higher in Huh 7.5 than PHESCs (Figure S1). Collectively these data suggested that PHESCs permitted HEV entry and replication. HEV replication was higher in PHESCs isolated from donor #1 than other donors, as shown by the viral load in the cell lysate and supernatant of the infected cells (Figure 2); therefore, we decided to complete the study using PHESCs from donor #1.

### 2.3. HEV Capsid Protein Was Assembled in PHESCs and the Virus Completes the Life Cycle in the Cells 

**Figure 3:**
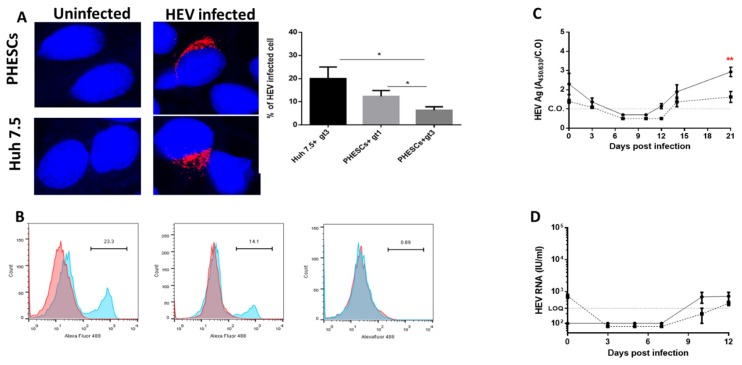
Assembly of HEV capsid protein and release of the virus from PHESCs. (**A**) Endometrium stromal cells (upper panel) or Huh7.5 cells (lower panel) either uninfected (left side) or infected with HEV preparations (right side) were fixed and stained with anti-ORF2 Ab (1-E6 Ab) (red). DAPI was used for nuclear staining (blue) (Scale bars, 20 µm.). The percentage of infection was determined in Huh 7.5 challenged with HEV-3 and PHESCs challenged with HEV-1 and HEV-3. Depicted are the mean values of three independent experiments ± SD. * *p* < 0.05, as determined by the unpaired t-test. (**B**) Flow cytometry shows the expression of HEV ORF-2 in PHESCs infected with HEV-1 (left) and HEV-3 (middle) and compared to mock-infected cells (right). Red histograms represent cells stained with the secondary A488-conjugated anti-mouse antibodies alone; blue histograms represent cells stained by mouse anti-HEV-ORF2 followed by A488-conjugated anti-mouse antibody. (**C**) Supernatants collected from HEV-1- (solid line) and/or HEV-3- (dotted line) infected PHESCs were tested for HEV ORF2 Ag by ELISA, C.O. is the cut off. The mean level of HEV ORF2 Ag in the supernatant collected at day 21 post-infection from HEV-1-infected PHESCs was compared with the level of HEV ORF2 Ag in the supernatant collected at the same time point from HEV-3-infected PHESCs. Depicted are the mean values of three independent experiments ± SEM. ** *p* < 0.01, as determined by the unpaired *t*-test. (**D**) Naive PHESCs were inoculated with cell lysate of HEV-1. HEV RNA load was quantified in these samples by qPCR. The solid line represents the intracellular HEV RNA load, the dotted line represents extracellular RNA load, and LOQ is the limit of quantification. Depicted are the mean values of three independent experiments ± SEM.

To examine the expression of HEV capsid protein in PHESCs, we infected PHESCs (from donor #1) with HEV-1 and/or HEV-3 for 14 days and then we assessed HEV ORF2 expression by immunofluorescence (IF) and flow cytometry. Huh 7.5 cells infected with HEV-3 served as a positive control. We detected HEV ORF2 inside the infected PHESCs by IF (Figure 3A). We assessed the percentage of infection in PHESCs; the mean percentage of infection in PHESCs challenged with HEV-1 and HEV-3 was 12.33 ± 1.45 and 6.333 ± 0.8819, respectively (Figure 3A). We confirmed the expression of HEV ORF2 Ag in PHESCs by flow cytometry (Figure 3B and Figure S2). 

To assess the viral assembly and release from PHESCs, the level of HEV ORF2 Ag was checked in the supernatants of HEV-infected cells by ELISA. Importantly, we found that the level of HEV ORF2 Ag increased over time, indicating the assembly and release of HEV particles from the infected cells (Figure 3C). In a parallel line to HEV RNA load, the level of HEV ORF2 Ag was significantly higher in the supernatant of HEV-1-infected PHESCs than the supernatant of HEV-3-infected PHESCs (Figure 3C). To confirm that complete infectious viral particles were released from PHESCs, cell-lysate-derived HEV-1, prepared from infected PHESCs on day 21, was used as inoculum to infect naive PHESCs. Intracellular and extracellular HEV RNA was detectable in these cells, and the viral loads increased with time, suggesting that the released viral particles from PHESCs were infectious (Figure 3D).

### 2.4. HEV-1 Upregulated the Inflammatory Transcriptome of PHESCs and Impaired Interferon (IFN) Type III Expression

**Figure 4:**
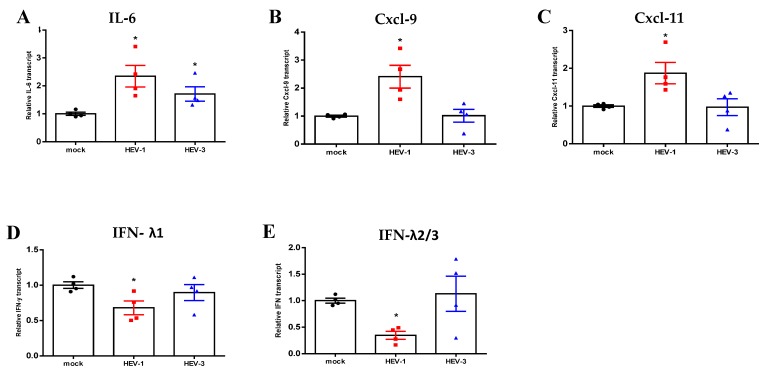
HEV-1 upregulated the inflammatory transcriptome and impaired the expression of Impaired Interferon (IFN) type III in PHESCs. PHESCs were challenged with HEV-1 and/or HEV-3 for 7 and 10 days, respectively, and then total RNA was extracted and the expression level of mRNA of proinflammatory markers (IL-6, Cxcl-9, and Cxcl-11) (**A**–**C**) and IFN transcripts (IFN-λ1 and IFN-λ2/3) (**D**,**E**) was normalized to the housekeeping gene (18srRNA). The relative gene expression was determined by comparing the expression levels of these transcripts with mock cells. The data represent the mean +/- SEM of four separate experiments. * indicates *p* ≤ 0.05, as assayed by a two-tailed Student’s *t*-test

Then, we asked if HEV infection affects the transcriptome of PHESCs. To verify this point, PHESCs were challenged with HEV-1 and/or HEV-3 for 7 and 10 days, respectively, and then total RNA was extracted and assessed for selected inflammatory and IFN transcripts. We analyzed the gene expression in PHESCs at the early time points when HEV RNA started to be detectable intracellularly. The intracellular RNA load was 700 and 500 IU/mL for HEV-1- and HEV-3-infected cells, respectively. Interestingly, we found that HEV-1 induced proinflammatory transcripts, such as Cxcl-9, Cxcl-11, and IL-6 transcript, while TNF-α, IL-β, MCP-1, Cxcl-10, IL-15, and IL-8 were not affected. On the other hand, HEV-3 infection only upregulated the IL-6 transcript and did not affect the expression of the other transcripts (Figure S3). Regrading IFN transcripts, HEV-1 downregulated type III IFN (IFN-λ1, IFN-λ2/3), while type I and II IFN (IFN-α, IFN-β, IFN-ɤ) were not changed (Figure S2). The expression of IFN transcripts was not altered by HEV-3 infection (Figure 4). 

### 2.5. Ribavirin (RBV) Abolished HEV Replication in PHESCs

**Figure 5:**
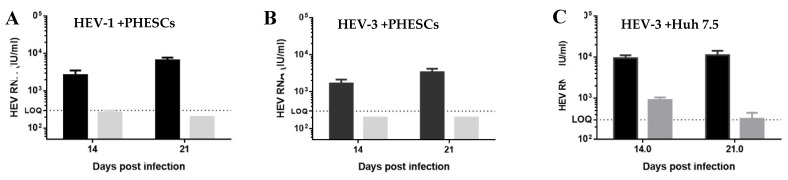
Ribavirin (RBV) abolished HEV replication in PHESCs. PHESCs, challenged with stool-derived HEV-1 (**A**) or stool-derived HEV-3 (**B**), were treated (grey) or not (black) with RBV (50 µM final conc). (**C**) Huh 7.5 infected with HEV-3 virus and treated (grey) or not (black) with RBV (50 µM final conc). The supernatants were collected from treated and untreated cells at 14- and 21-days post-infection, and HEV RNA was quantified in these samples by qRT-PCR. Depicted are the mean values of three independent experiments ± SEM.

As a proof of concept for HEV replication in PHESCs, we assessed the effect of RBV on HEV replication in PHESCs. We treated PHESCs and Huh 7.5 with RBV at a dose of 50 µM (final concentration) after challenging with HEV preparations and then we monitored the viral load in the supernatants collected from treated and non-treated cells at day 14 and 21 post-infection by qRT-PCR. We found that RBV treatment inhibited the replication of HEV-1 and HEV-3 in PHESCs and in Huh 7.5 cells (Figure 5), as shown by the decrease in HEV RNA titers in the supernatants of the treated cells compared to untreated.

## 3. Discussion

HEV causes acute, chronic and extrahepatic manifestations. Several extrahepatic manifestations have been reported in association with HEV infection, such as neurological disorders, glomerulonephritis, cryoglobulinemia, acute pancreatitis, thrombocytopenia, and hemolytic anemia [21,31]. The extrahepatic manifestations are mediated by either direct HEV replication in these tissues, or indirectly by various immune-mediated mechanisms [21]. The pathogenesis of HEV in the genital system is not largely understood. HEV-4 could disrupt the blood–testis barrier in the Mongolian gerbils’ model and BALB/c mice, inducing the necrosis of tubules, germ cell apoptosis, and testicular damage [22,32]. In addition, HEV RNA was detected in pig semen in Shaanxi Province, China, and the semen of infertile males [23,33]. While others reported that there is no link between male infertility of HEV-3 infection [34]. Still little is known about the pathogenesis of HEV in the female reproductive tract (FRT). An et al. reported the replication of HEV-4 in the ovary of the rabbits promoting oocyte apoptosis and the infected ovary could be a source for HEV vertical transmission [24]. Besides, Yang et al. reported that HEV replicates in the uterus of pregnant BALB/c mice, suggesting that the replication of HEV in the uterus could be a cause of vertical transmission of HEV during pregnancy [25]. Gouilly et al. reported that HEV targets stroma cells derived from decidua and placenta at the maternal–fetal interface, suggesting that HEV replication in these cells facilitates the vertical transmission mechanism of HEV similar to the TORCH pathogens [29]. A higher viral load in the plasma and the deregulation of progesterone receptors are associated with poor pregnancy outcome and facilitate the exposure of the maternal–fetus interface to the circulating HEV particles in the plasma [18]. During pregnancy, the fibroblast-like stromal cells are differentiated to rounded, epithelioid-like cells (decidualized endometrial stromal cells), and this process is called decidualization. The decidualization is a complex process derived by an interplay of sex hormones, transcription factors, cytokines, and signaling pathways, and it occurs in pregnancy and secretory phase of menstruation to maintain the embryo implantation [35]. Herein, we showed that HEV replicates and completes the life cycle in fibroblast endometrial stromal cells isolated from non-pregnant women, i.e., non-decidualized endometrial stromal cells, and this is the new finding of our study. 

In this study, we showed that HEV RNA and HEV ORF2 Ag were increased over time in PHESCs challenged with HEV preparations, indicating efficient replication in vitro and the life cycle of HEV is completed in these cells. Similar kinetic in HEV RNA and capsid proteins were reported in other cell lines that support HEV replication and represent sites of extrahepatic HEV replication, such as neuron and placenta-derived cell lines [36,37]. HEV-1 replicates more efficiently in PHESCs than HEV-3. A higher viral load means more accumulated extracellular capsid protein and a higher percentage of HEV-infected cells were observed in HEV-1-infected cells. Similar findings were reported by Gouilly et al. who showed that the replication of HEV-1 was significantly higher than the HEV-3 in the maternal decidua, fetal placenta, and the placental stromal cells, producing more infectious progeny virions [29]. We did not observe any microscopic morphological changes in PHESC infected with HEV, suggesting that HEV is not cytotoxic. Similarly, HEV is not cytopathic to primary placental stromal cells [29] and not hepatotoxic [38,39,40].

Comparing the replication of HEV-3 in PHESCs versus Huh 7.5 cells, we found that HEV replication was significantly higher in Huh 7.5 cells than PHESCs, as shown by a higher viral load in the supernatant of Huh 7.5 and a higher percentage of infected cells. Since the main site of HEV replication is the liver, it is expected that the replication of HEV in the hepatocyte-derived cell line is higher than its replication in other sites. Our result agreed with Drave et al., who showed that the replication of the HEV-3 cell culture adapted virus (P6 Kernow C1 strain) was higher in the hepatocyte-derived cell line (HepG2 cells) than the neuron-derived cell lines [36]. Similarly, Gouilly et al. reported that the replication of HEV-3 was higher in HepG2 cells than in the explants derived from the decidua and placenta [29].

We showed here that HEV infection changes the transcriptome of PHESCs; HEV infection upregulates the inflammatory transcripts, such as CXCl-9, CXCL-11, and IL-6. In addition, IFN type III was impaired in PHESCs infected with HEV-1. Our results agreed with Gouilly et al., who showed that HEV-1 induced tissue damage at the maternal–fetus interface by altering the secretome and increasing the release of proinflammatory cytokines, such as IL-6 [29]. Other studies showed that Cxcl-9 and CXCl-10 were significantly upregulated in human liver chimeric mice infected with the HEV-1 sar55 strain [39]. Moreover, HEV-1 infection impairs IFN signaling pathways at the maternal–fetus interface to allow the virus to replicate and spread [29]. HEV-1 causes most of the complications associated with HEV infection during pregnancy, such as abortion, premature delivery, the death of a liveborn baby soon after birth or fetal and/or maternal mortality. While the course of HEV-3 during pregnancy is a mild to moderate, spontaneously self-limited [19,21].

To our knowledge, our study is the first report that shows HEV replication in human endometrial stromal cells in the absence of pregnancy. Endometrial stromal cells are permissive to other viruses, such as HIV, Human Herpes, and Zika virus [27,28]. Neidleman et al. showed that endometrial stromal cells significantly increased the infection of HIV to CD4+ T cells by 37-100-folds, and these cells play a role in HIV acquisition at the mucosal sites [28]. Moreover, Pagani et al. reported the replication of the Zika virus in endometrial stromal cells and they hypothesized that the presence of this virus in the female genital system could foster the vector independent mode of transmission (either the vertical transmission from mother to fetus or the sexual transmission from female to male) [27]. Likewise, the PHESCs could be a reservoir for HEV, and these cells could be an endogenous source for the vertical HEV transmission during the pregnancy since these cells are morphologically and functionally changed to develop the decidua, under the effect of progesterone, which facilitates the placenta formation in case of implantation. The pathogenesis and impact of HEV infection in the female genital system, especially in the absence of pregnancy, are unknown. Recent studies showed that HEV infection could cause testicular damage, germ cell apoptosis, and male infertility [22,23]. We showed here that HEV-1 induced the inflammatory response in PHESCs. Further studies should be done to assess the pathogenesis, clinical symptoms, and outcomes of HEV infection in the female genital system, especially in the absence of pregnancy. 

## 4. Materials and Methods 

### 4.1. Isolation and Characterization of PHESCs from Non-Pregnant Women

PHESCs were isolated from proliferative human endometrium collected from healthy non-pregnant women during the proliferative stage of menstruation (n = 5), aged 27–35 years without a history of gynecological abnormalities, as described previously [30] in Table 1. Briefly, endometrial biopsies were washed with phosphate-buffered saline (PBS), minced into 1–2-mm pieces and then digested by 0.1% collagenase type I for 2 h in a shaking water bath at 37 °C. PHESCs were purified and separated using 40 and 20 µm sieves filters. The filtrate was centrifuged, and the cell pellet was dissolved in DMEM-F12 media (Gibco, ThermoFisher Scientific) supplemented with 10% fetal bovine serum (Gibco, ThermoFisher Scientific), and cultured at 37 °C and 5% CO_2_. PHESCs at passages 2–6 were used in the infection experiments before they showed signs of senescence. For the characterization of PHESCs, cells were stained with anti-human CD90 FITC-conjugated antibodies (Novus Biologicals; Centennial, CO, USA), anti-human Vimentin Alexa Fluor^®^ 488-conjugated monoclonal antibodies (R&D Systems; Minneapolis, MN, USA) or anti-cytokeratin Alexa Fluor^®^ 488-conjugated monoclonal antibodies (ThermoFisher; Waltham, MA, USA). Isotype matched antibodies were used as staining controls. The cells were acquired using the FACSC alibur flow cytometer (BD Biosciences, USA) and the data were analyzed using FlowJo software. All donors tested negative for viral hepatitis markers (HAV, HBV, HCV, and HEV markers) at the time of biopsies collection and/or PHESC isolation. All participating women provided written informed consent, and the use of human endometrial tissue samples was approved by the Institutional Review Board (IRB) at the Faculty of Medicine, Assiut University, Egypt, in accordance with the provisions of the Declaration of Helsinki.

### 4.2. HEV Inoculums

HEV inoculums were isolated from the stool of acute HEV-infected patients admitted for diagnostic purposes to Assiut University hospitals. The stool was dissolved in PBS to prepare 10% *w*/*v* fecal suspension, then centrifuged, and the supernatant was filtered using a 0.22-μm filter (Millipore) and used as inoculum for the infection experiments. The viral load of the inoculums was quantified by qPCR, as described in detail in the Section 4.4. The sequencing and phylogenetic analysis of the isolated viruses was done as previously described and according to Smith et al. [4,39,41].

### 4.3. Infection of PHESCs with HEV Preparations

One day prior to infection, 2 × 10^4^ cells/well of PHESCs were seeded in a 24-well plate. A filtered 10% *w*/*v* fecal preparation was added to the cells at a dose of 10^6^ IU/well and incubated for 6 h. After that, the inoculum was removed and replaced with fresh culture medium. Huh 7.5 cells (kindly provided by VACSERA-Cell Culture Unit, Cairo) infected with the same inoculums were used as a positive control. The lysis of the cells and collection of supernatants were done at 0, 1, 3, 5, 7, 10, 12, 14, and 21 days post-infection. The cell lysate and supernatant from each time point were saved at −80 °C, and processing of all samples was done at the same time. HEV-1 cell lysate was prepared as described previously to be used as inoculum in infectivity experiment [39]. For infectivity assay, PHESCs were seeded in a 96-well plate and challenged with HEV-1 cell lysate. HEV RNA load was quantified by qPCR. 

### 4.4. Quantification of HEV RNA by qRT-PCR

The quantification of HEV RNA was done as described previously with slight modifications in the procedure [38,39,41,42]. Briefly, viral RNA was extracted from 10% *w*/*v* stool preparations and cell culture supernatants using a QIAamp Viral RNA Mini Kit (Qiagen, Germany) and from the HEV-infected cells using an RNeasy Mini Kit (Qiagen, Germany) according to the manufacturer’s instructions. HEV RNA was quantified using primers targeting HEV ORF3 as described before [38,39,42]. The limit of quantification (LOQ) of our assay is 300 IU/mL for undiluted samples. 

### 4.5. Detection of HEV ORF2 Ag in the Infected PHESCs by Immunofluorescence and Flow Cytometry

PHESCs cells were challenged with the stool-derived HEV-1 and/ or HEV-3 as described in the previous section. After 14 days, the cells were fixed using cold methanol, permeabilized by 0.5% Triton X100 and stained with antibody 1E6 clone (Millipore; dilution 1/500 for IF). Goat anti-mouse IgG conjugated with Alexa647 (Invitrogen) was used as a secondary antibody (dilution 1/2000 for IF) and DAPI was used for nuclei staining. For flow cytometry, the cells were fixed and permeabilized using eBioscience™ Fixation/Permeabilization Concentrate (ThermoFischer Scientific, CA, USA) according to the manufacturer’s instruction and then stained with antibody 1E6 clone (Millipore; dilution 1/1000) that targets the HEV ORF2 protein. Goat anti-mouse IgG conjugated with Alexa488 (Invitrogen) was used as a secondary antibody according to the manufacturer’s instructions. Uninfected PHESCs processed by the same procedure were used as a negative control. 

### 4.6. Monitoring of Extracellular HEV Capsid Protein by ELISA

Detection of HEV ORF2 Ag in cell culture supernatants was performed using the HEV-Ag ELISA^Plus^ assay (Bejing Wantai Biological Pharmaceutical Co., Beijing, China) according to the manufacturer’s instructions, with slight modifications in the procedure of cut-off (C.O.) calculation, as described previously [40].

### 4.7. Test the Effect of HEV Infection on the Transcriptome of PHESCs 

Total cellular RNA was extracted PHESCs using the RNeasy Mini Kit (Qiagen, Hilden, Germany) with on-column DNase treatment according to the manufacturer’s instructions. RNA was converted into complementary DNA (cDNA) using MultiScribe reverse transcriptase according to the manufacturer’s instructions (Invitrogen, California). Quantitative real-time polymerase chain reaction (qPCR) was carried out using SYBR green master mix (Applied Biosystems, Foster City, California, USA) on 7500 Fast Real-Time PCR (Applied Biosystems) for target genes and normalized to the housekeeping gene (18srRNA) using the 2^-ΔΔCt^ method. The sequences of primers used in this study are listed in Table 2.

### 4.8. Testing the Effect of Ribavirin (RBV) on HEV Replication in PHESCs

PHESCs were treated with 50 µM of RBV (Sigma-Aldrich, Darmstadt, Germany) after HEV infection as described previously [29,36,37] with a slight modifications in the procedure. Briefly, 2 × 10^4^ cells/well were seeded in a 24-well plate and were incubated at 37 °C. After 24 h, culture medium was removed, and the cells were challenged with HEV preparations (10^6^ IU/mL) with or without RBV. After 6 h, the inoculum was removed, the cells were washed, and 1 mL medium with or without RBV was added. The supernatants were collected at 0, 14, and 21 days and stored at −80 °C until analysis. Huh 7.5 cells infected with stool-derived HEV-3 virus and treated with RBV (50 µM) served as a control. The quantification of HEV RNA in the supernatants was done by qRT-PCR as described previously.

### 4.9. Statistics

Statistical analyses were performed using the GraphPad Prism software 6 (GraphPad Software, La Jolla, CA, USA) using the unpaired *t*-test. *p* < 0.05 was considered significant.

## Figures and Tables

**Table 1:** Age of the women enrolled in the study and screening tests done on the samples.

Donor #	Age	Screening Tests ^1^
1	27	HAV: rapid tests for anti-HAV IgM
2	32	HBV: HBsAg and anti-HBV core IgM
3	34	HCV: anti-HCV IgG.
4	35	HEV: Anti HEV IgM, anti HEV IgG, HEV Ag and
5	33	HEV RNA

^1^ All participants tested negative for viral hepatitis markers at the time of sample collection.

**Table 2:** Target genes and sequences of primers used in qPCR analysis.

Gene	Primer Sequence 5′-3′	Product Size (bp)
18srRNA	Forward GTAACCCGTTGAACCCCATT Reverse CCATCCAATCGGTAGTAGCG	151
IL-6	Forward TCAATATTAGAGTCTCAACCCCCA Reverse TTCTCTTTCGTTCCCGGTGG	90
IL-8	Forward ATGACTTCCAAGCTGGCCGTGGCT Reverse TCTCAGCCCTCTTCAAAAACTTCTC	292
IL-15	Forward: CTGACGTCACATGGAGCACA Reverse: CTGCACTGAAACAGCCCAAA	283
IL- β1	Forward CCACAGACCTTCCAGGAGAATG Reverse GTGCAGTTCAGTGATCGTACAGG	131
MCP-1 (CCL2)	Forward AGTCTCTGCCGCCCTTCT Reverse GTGACTGGGGCATTGATTG	93
TNF-α	Forward CGCTCCCCAAGAAGACAG Reverse AGAGGCTGAGGAACAAGCAC	60
Cxcl-9	Forward AGTGCAAGGAACCCCAGTAG Reverse AGGGCTTGGGGCAAATTGTT	112
Cxcl-10	Forward: CCACGTGTTGAGATCATTGCT Reverse: TGCATCGATTTTGCTCCCCT	152
Cxcl-11	Forward: GAGTGTGAAGGGCATGGCTA Reverse: ACATGGGGAAGCCTTGAACA	71
IFN-α	Forward: CCTGATGAATGCGGACTCCA Reverse: TAGCAGGGGTGAGAGTCTTTG	265
IFN-β	Forward: CGCCGCATTGACCATCTA. Reverse: GACATTAGCCAGGAGGTTCTC.	112
IFN-ɤ	Forward GAGTGTGGAGACCATCAAGGAAG Reverse TGCTTTGCGTTGGACATTCAAGTC	124
IFN-λ1	Forward: GCAGGTTCAAATCTCTGTCACC Reverse AAGACAGGAGAGCTGCAACTC	109
IFN-λ2/3	Forward: CAGCTGCAGGTGAGGGA Reverse GCGGTGGCCTCCAGAACCTT	77

## Supplementary Materials

The following are available online at https://www.mdpi.com/2076-0817/9/4/295/s1, Figure S1: Replication of HEV inoculums in Huh 7.5 cells, Figure S2: Flow cytometry shows the expression of HEV ORF-2 in PHESCs; Figure S3: Effect of HEV infection on the transcriptome of PHESCs.
