# Supplementary material for: Evidence of the Extrahepatic Replication of Hepatitis E Virus in Human Endometrial Stromal Cells
Source: Pathogens. 2020 Apr 17;9(4):295. doi: 10.3390/pathogens9040295 (PMC7238207; doi:10.3390/pathogens9040295)
Supplement: Supplementary file 1 [file pathogens-09-00295-s001.pdf]

**Supplementary Materials:**

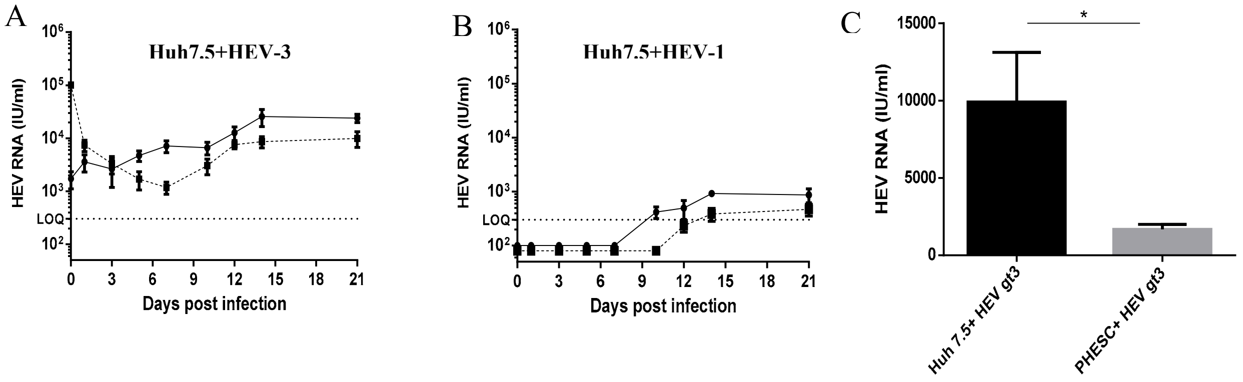


**Figure S1.** Replication of HEV inoculums in Huh 7.5 cells. Huh 7.5 cells were challenged with stool derived HEV-3 (A) or stool derived HEV-1 (B), then lysis of cells and/or collection of supernatants were done at 0, 1, 3, 5, 7, 10, 12, 14- and 21-days p.i. HEV RNA load was quantified in these samples by RT-qPCR. Solid line represents intracellular HEV RNA load, dotted line represents extracellular RNA load and LOQ is the limit of quantification. (C) The titer of HEV RNA in the supernatant collected at day 21 from HEV-3 infected Huh7.5 cells was compared with the titer of HEV RNA in the supernatant collected at the same time point from HEV-3 infected PHESCs. * indicates $P \leq 0.05$, as assayed by a two-tailed Student's t-test.

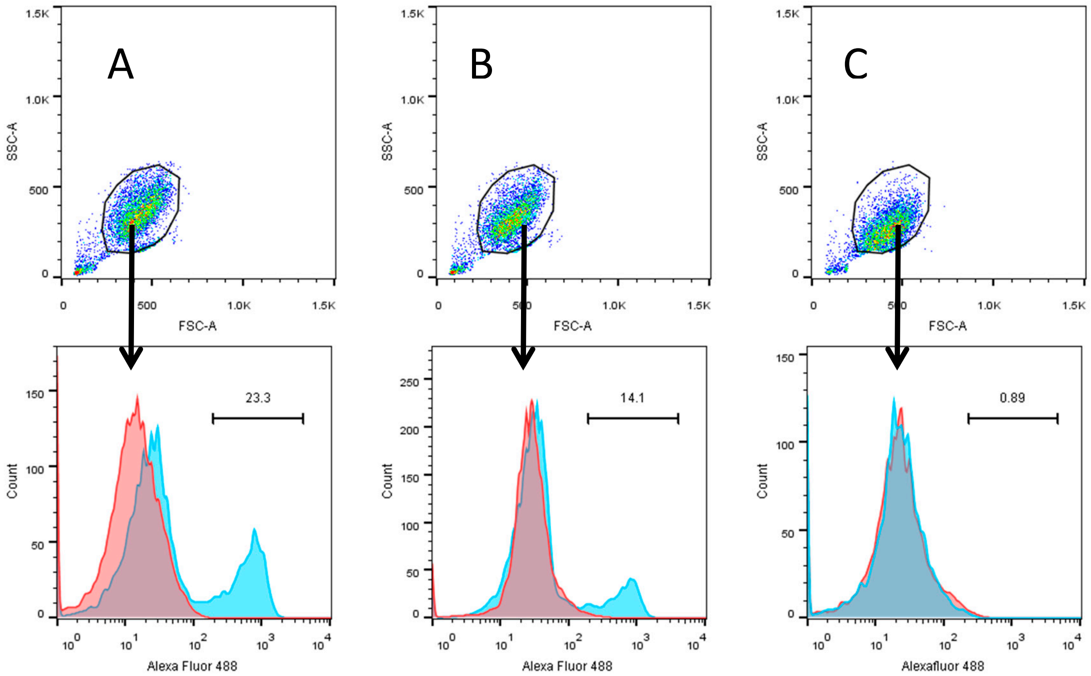


**Figure S2.** Flow cytometry shows the expression of HEV ORF-2 in PHESCs. PHESCs infected with HEV-1 ( A) and HEV-3 ( B) and compared to mock-infected cells (C). Cells were selected based on their forward scatter/side scatter dot plot diagrams; Red histograms represent cells stained with the secondary A488 conjugated anti-mouse antibodies alone; blue histograms represent cells stained by mouse anti-HEV-ORF2 followed by A488 conjugated anti-mouse antibody.

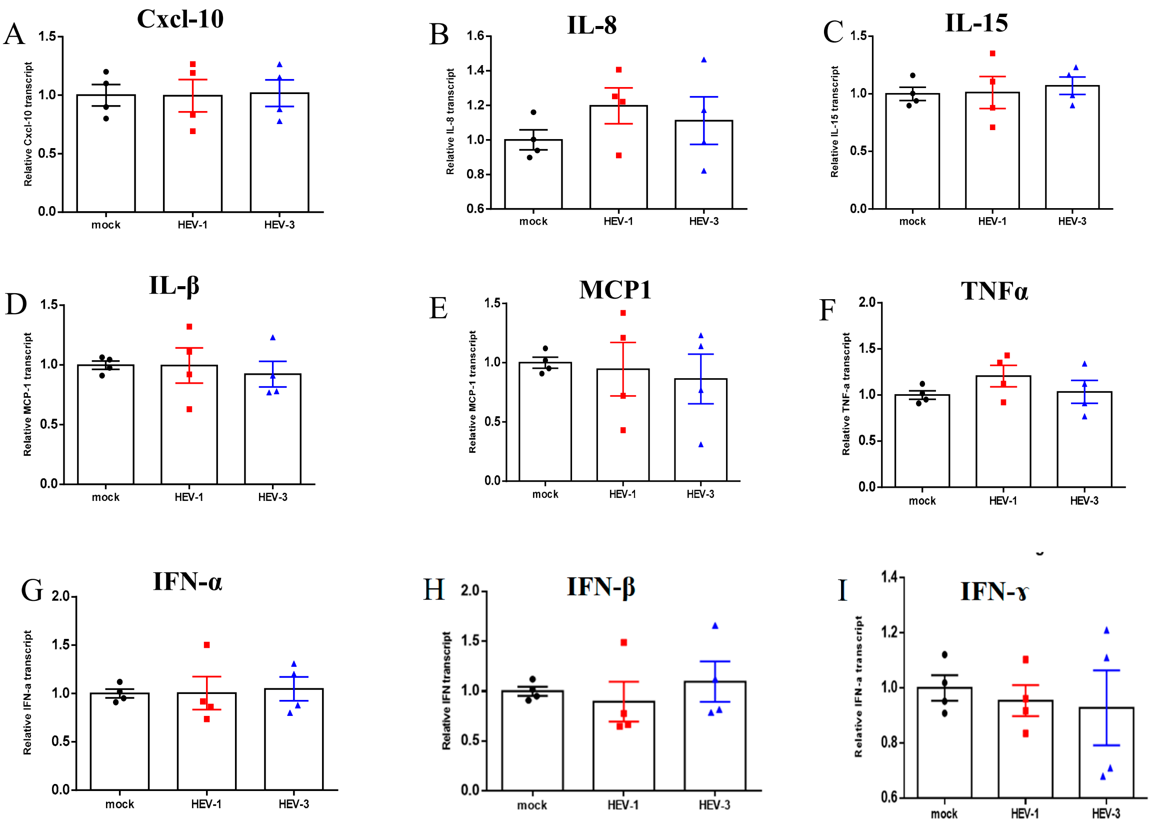


**Figure S3.** Effect of HEV infection on the transcriptome of PHESCs. PHESCs were challenged with HEV-1 and/or HEV-3 for 7 and 10 days respectively and then total RNA was extracted and the expression level of mRNA of proinflammatory markers (IL-8, IL-15, Cxcl-10, MCP-1, TNF-$\alpha$ and IL-β) (A–F) and IFN transcripts (IFN-$\alpha$, IFN-β and IFN-ɤ) (G-I) was normalized to the housekeeping gene (18s rRNA). The relative gene expression was determined by comparing the expression levels of these transcripts with mock cells. Data represent the mean +/- SEM of four separate experiments.
